# Depth-dependent microbial metagenomes sampled in the northeastern Indian Ocean

**DOI:** 10.1038/s41597-024-02939-4

**Published:** 2024-01-18

**Authors:** Xiaomeng Wang, Muhammad Zain Ul Arifeen, Shengwei Hou, Qiang Zheng

**Affiliations:** 1 State Key Laboratory of Marine Environmental Science, College of Ocean and Earth Sciences, Institute of Marine Microbes and Ecospheres, Xiamen University, Xiamen, 361102 PR China; 2 Fujian Key Laboratory of Marine Carbon Sequestration, Xiamen University, Xiang’an Campus, Xiang’an South Road, Xiamen, 361102 China; 3 Department of Ocean Science and Engineering, Southern University of Science and Technology, Shenzhen, 518000 China

**Keywords:** Microbial ecology, Biodiversity, Marine biology

## Abstract

The northeastern Indian Ocean exhibits distinct hydrographic characteristics influenced by various local and remote forces. Variations in these driving factors may alter the physiochemical properties of seawater, such as dissolved oxygen levels, and affect the diversity and function of microbial communities. How the microbial communities change across water depths spanning a dissolved oxygen gradient has not been well understood. Here we employed both 16S rDNA amplicon and metagenomic sequencing approaches to study the microbial communities collected from different water depths along the E87 transect in the northeastern Indian Ocean. Samples were collected from the surface, Deep Chlorophyll Maximum (DCM), Oxygen Minimum Zone (OMZ), and bathypelagic layers. Proteobacteria were prevalent throughout the water columns, while Thermoproteota were found to be abundant in the aphotic layers. A total of 675 non-redundant metagenome-assembled genomes (MAGs) were constructed, spanning 21 bacterial and 5 archaeal phyla. The community structure and genomic information provided by this dataset offer valuable resources for the analysis of microbial biogeography and metabolism in the northeastern Indian Ocean.

## Background & Summary

The Indian Ocean is bordered by the Southern Ocean to the south and enclosed by continental shelf and land masses on other sides, covering approximately 20% of the global surface ocean. The hydrographic characteristics of the Indian Ocean are influenced by a multitude of geological and physicochemical processes, including tectonic activities^1^, oceanic circulation patterns^2^, boundary currents^3^, climate modes^4^, and land-ocean interactions^5^, etc. Three distinct biomes have been proposed based on the biogeochemical characteristics of the Indian Ocean^6^, including the oligotrophic subtropical southern Indian Ocean, the iron-deficient low-productivity equatorial region, and the nutrient-rich high-productivity northern Indian Ocean^6–8^. The northern Indian Ocean, particularly the Bay of Bengal (BoB), also receives significant freshwater discharge^9,10^ and atmospheric deposition^11^, which increase surface productivity and strengthen stratification. In conjunction with the limited oxygen supply from deep overturning circulation and lateral advection in the northern Indian Ocean^2^, the mid-depth waters ranging approximately from 200 to 1000 m are oxygen deficient to a vast extent, forming two large oxygen minimum zones (OMZs) in the Arabian Sea and the Bay of Bengal^12^. Collectively, the areas covered by OMZs in the Indian Ocean account for more than half of the global OMZs (59%)^13^, with the Bay of Bengal standing as the world’s largest hypoxic bay^14^. Despite significant seasonal variations in monsoon winds and biological productivity, dissolved oxygen concentrations in these regions exhibit relatively minor fluctuations^15^.

Marine microorganisms play a central role in driving various elemental cycles within the global ocean due to their high abundance, immense diversity, and versatile metabolic capacity^16,17^. The Indian Ocean has a great influence on global biogeochemical cycles by contributing around 15% of oceanic net primary production^18^, with a particularly higher abundance of picocyanobacteria than most other oceanic basins^19^. Dissolved oxygen is one of the most important factors controlling microbial respiration and biogeochemical transformation in marine environments^20^. In oxygen-deficient waters, alternative electron acceptors such as nitrate were used or preferred by diverse marine organisms^21,22^. OMZs are characterized by significant REDOX gradients, and the nitrogen cycle dominates the biogeochemical processes^23,24^. The continuous expansion of marine OMZs will be accompanied by more widespread anammox and denitrification activities, which will have a profound influence on nitrogen bioavailability in marine environments^25^. To better understand the role of biological communities within OMZs, it is important to study their diversity, metabolic function, and ecological relationships^26,27^.

**Fig. 1:**
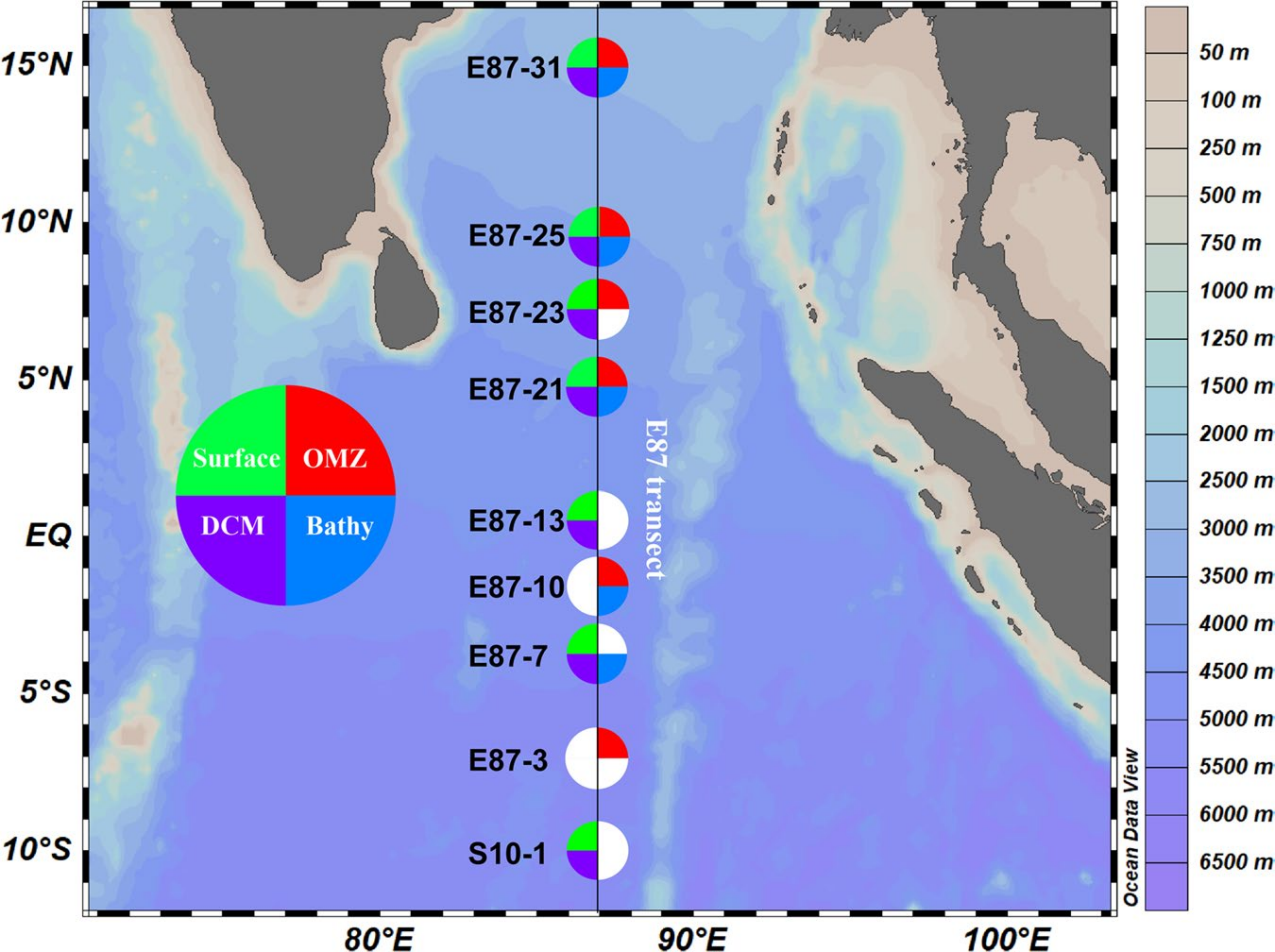
Sampling sites and layers along the E87 transect in the northeastern Indian Ocean. Surface, the surface layer at 5 m. DCM, the Deep Chlorophyll Maximum layer. OMZ, the oxygen minimum zone layer. Bathy, the bathypelagic layer at 2000 m. Detailed sample metadata can be found in Table S1.

**Fig. 2:**
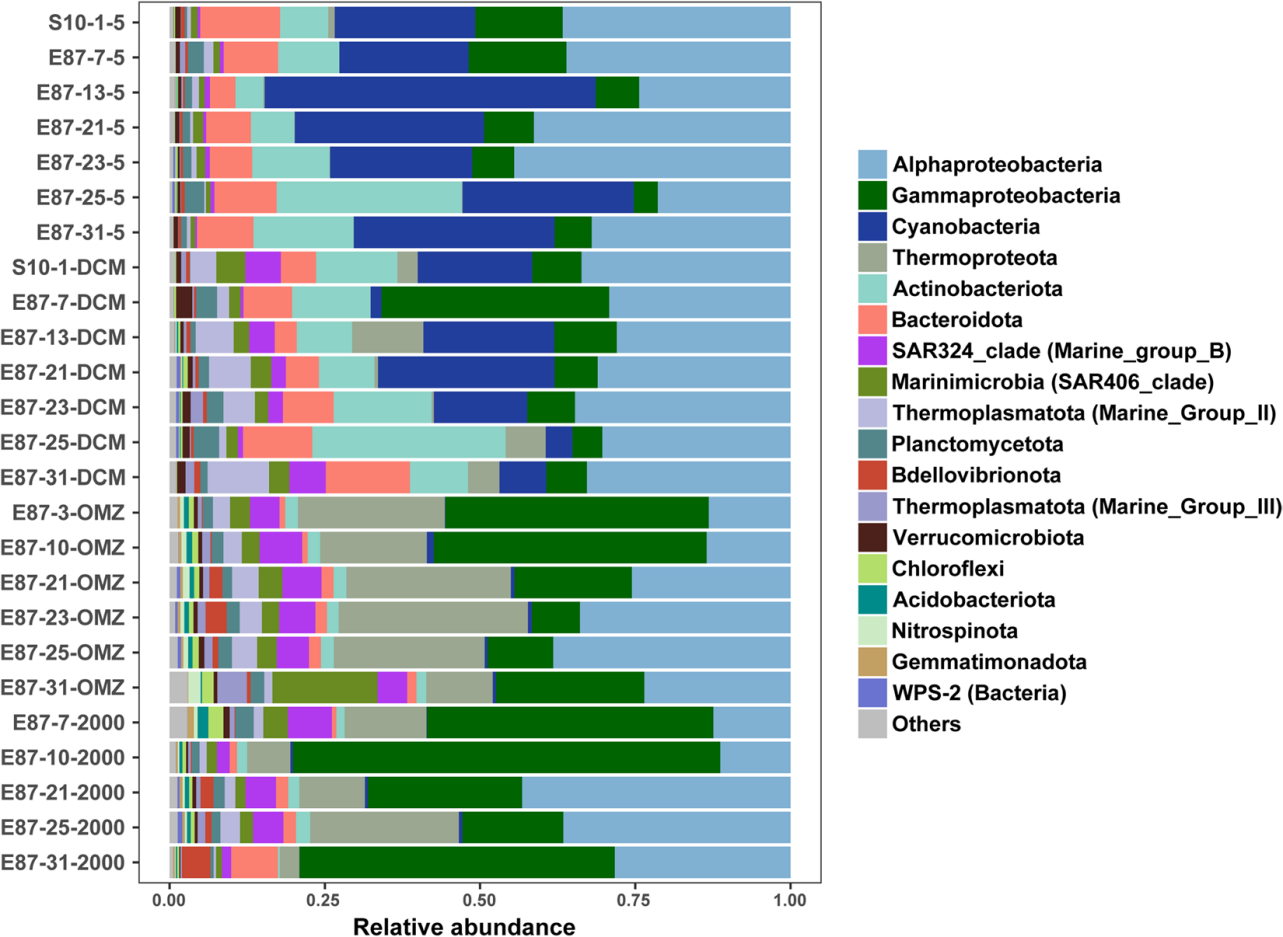
The relative abundance of different taxa across depths based on 16S rDNA amplicon sequencing in the northeastern Indian Ocean. Amplicon sequences were denoised and grouped into Amplicon Sequence Variants (ASVs) to calculate microbial relative abundance in each sample. Detailed 16S rDNA taxonomy assignment can be found in Table S2.

In this study, we conducted a comprehensive sampling expedition from April 15^th^ to June 20^th^, 2020, along the E87 transect in the Northeast Indian Ocean, spanning from 10°S off the East India coast to 15°N in the BoB. A total of 25 water samples were collected from various depths, including the surface (5 m, n = 7), DCM (n = 7), OMZ (n = 6), bathypelagic (Bathy) layers (2000 m, n = 5), for studying microbial diversity and metabolic potentials (Fig. 1). Detailed sample metadata including geographic locations and environmental factors can be found in Table S1. Flow cytometry analysis showed that the abundance of *Prochlorococcus* and picoeukaryotes reached their maxima in the DCM layer. In contrast, a higher abundance of *Synechococcus* was observed near the surface (Table S1). The 16S rDNA amplicon data revealed that Proteobacteria constituted the dominant phylum, accounting for 49.31% of all reads. Within Proteobacteria, Alphaproteobacteria accounted for 59.72%, while Gammaproteobacteria represented 29.44%. Notably, Gammaproteobacteria dominated in both the OMZ and Bathy waters. Cyanobacteria, on the other hand, were primarily distributed in the DCM and higher layers, accounting for 12.46% of all reads. Thermoproteota (Marine Group I archaea, MGI) emerged as a significant component of the OMZ layer, accounting for 8.77% of all reads. MGII (Marine Group II archaea) was predominantly found in the DCM, and although the relative abundance of MGIII (Marine Group III archaea) was relatively low across the water column, it was significantly higher in the OMZ layer compared to other layers (Fig. 2 and Table S2).

**Fig. 3:**
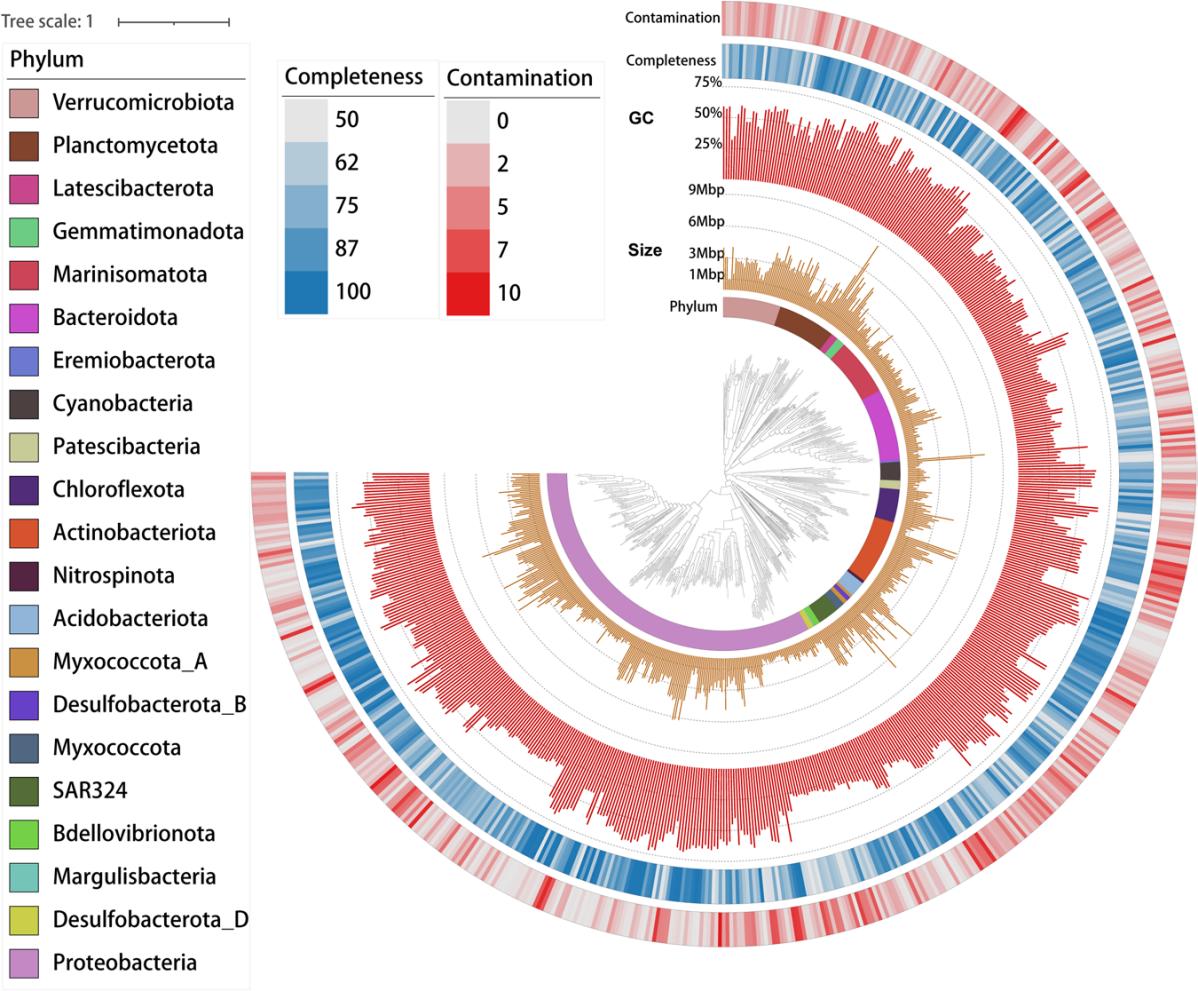
The phylogenomic tree of 571 bacterial MAGs reconstructed from the northeastern Indian Ocean. The universally conserved 160 single-copy marker genes were used to build this maximum-likelihood phylogenomic tree with 1000 bootstraps. Detailed MAG taxonomy assignment, associated with completeness and contamination information can be found in Table S2.

**Fig. 4:**
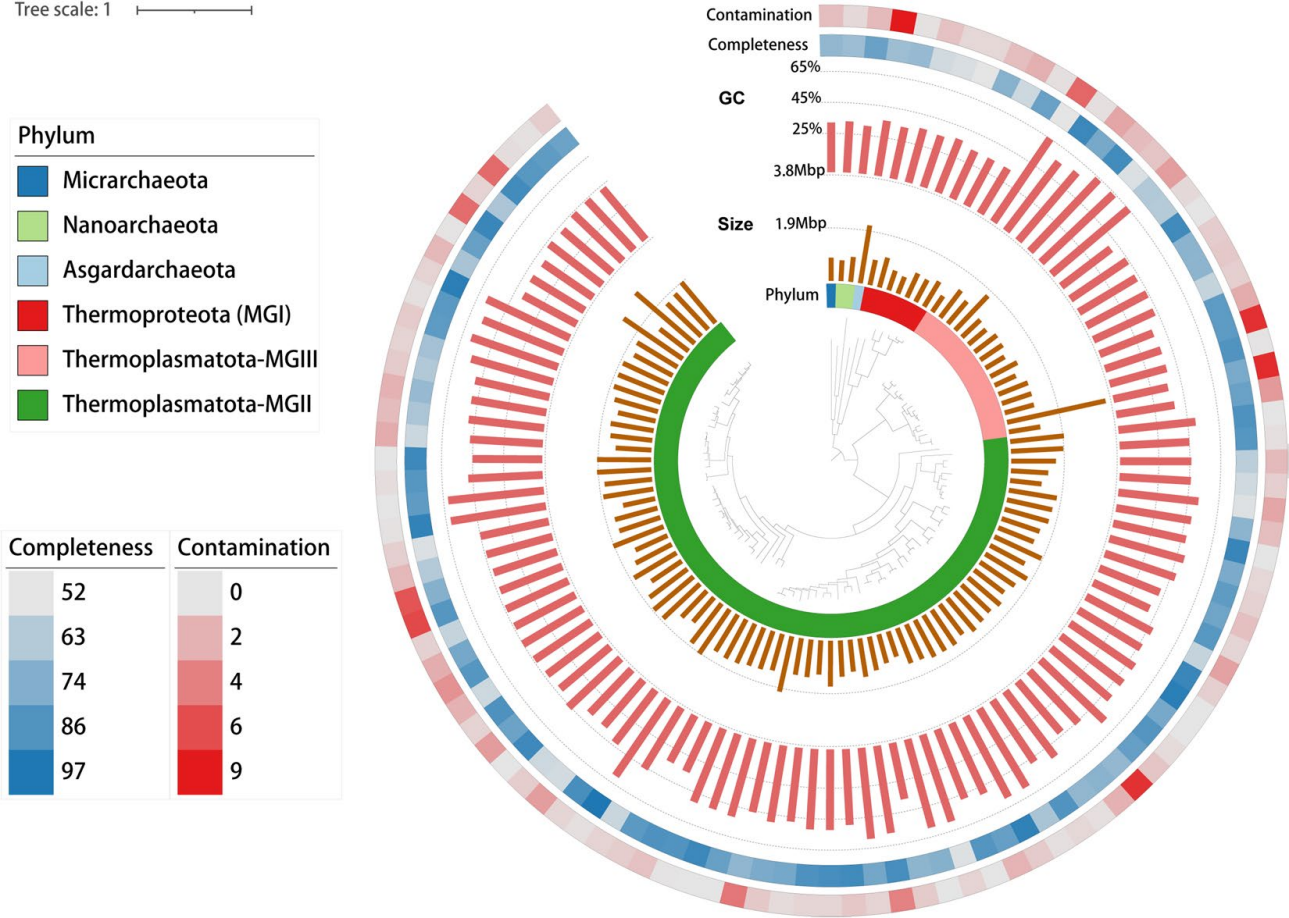
The phylogenomic tree of 104 archaeal MAGs reconstructed from the northeastern Indian Ocean. The universally conserved 49 single-copy marker genes were used to build this maximum-likelihood phylogenomic tree with 1000 bootstraps. Detailed MAG taxonomy assignment, associated with completeness and contamination information can be found in Table S2.

After metagenomic binning and refinement, a total of 675 non-redundant metagenome-assembled genomes (MAGs) with completeness ≥50% and contamination ≤10% were recovered, covering 21 bacterial and 5 archaeal phyla (Figs. 3, 4). Based on the MIMAG (Minimum Information about a Metagenome-Assembled Genome) standards^28^, 164 of these MAGs were classified as high-quality (completeness >90% and contamination <5%), accounting for 24.3% of the total MAGs. Compared with MAGs of OceanDNA^29^ and Tara Oceans^30^ at the identity threshold of 95%, we found that 62.45% of the MAGs reported here were not covered by either dataset, suggesting the uniqueness of MAGs recovered from the northeastern Indian Ocean. These MAGs were taxonomically classified into 104 archaeal and 571 bacterial genomes based on Genome Taxonomy Database (GTDB) release r207^31^. Bacterial phyla with >10 MAGs include Proteobacteria (n = 251), Bacteroidota (n = 50), Actinobacteriota (n = 45), Marinisomatota (n = 40), Planctomycetota (n = 40), Verrucomicrobiota (n = 40), Chloroflexota (n = 23), SAR324 (n = 15), Cyanobacteria (n = 13), and Acidobacteriota (n = 11) (Fig. 3 and Table S2). Archaeal phyla include Thermoplasmatota (n = 93), Thermoproteota (n = 7), Nanoarchaeota (n = 2), Asgardarchaeota (n = 1), and Micrarchaeota (n = 1) (Fig. 4 and Table S2). MAGs of MGIII archaea formed two distinct phylogenetic clusters with divergent GC contents, as previously reported^32^ (Fig. 4 and Table S2).

Complementary to the MAG-based analysis, genes were called on the contig level to construct a community-level gene catalog. After gene calling and deduplication, a total of 9,908,058 unique genes were recovered and function annotated with KEGG Orthology (KO) groups. The relative abundance of each unique gene in each sample was calculated in RPKM values. Gene sequences and a table of gene abundance across samples with functional annotations were provided (see the “Data records” section).

## Materials and Methods

### Sample collection and preparation

Samples were collected from the Northeast Indian Ocean, spanning latitude 10°S to 15°N along longitude 87°E, during the R/V “Shiyan3” cruise from April 15 to June 20, 2020 (Fig. 1). A total of 25 seawater samples were collected from 9 distant sites, covering both surface waters and deeper ocean regions. Fifteen liters of seawater were pre-filtered using a 20 μm nylon mesh (Sefar Nitex, Sweden), followed by subsequent filtration through a 0.22 μm pore size polycarbonate filter (Millipore, MA, USA). The filters were frozen in liquid nitrogen onboard and kept at −20 °C until DNA extraction. For microbial abundance estimation, 2 mL seawater samples were first filtered through a 20 μm nylon mesh, then fixed with 1% (vol/vol) glutaraldehyde, incubated in the dark for 15 minutes, and promptly frozen in liquid nitrogen and preserved at −20 °C for subsequent analysis. *In-situ* measurements of water temperature, salinity, dissolved oxygen (DO), and fluorescence were conducted using conductivity-temperature-depth (CTD) oceanic profilers (SBE-911 Plus). Other chemical parameters, including nitrite nitrogen, nitrate nitrogen, phosphate, and silicate concentrations were assessed using the Technicon AA3 Auto-Analyzer (Bran-Luebbe, Germany)^33^. Samples were named following the pattern of “station_name-water_depth”. For instance, the sample name “S10-1-5” indicates this sample was taken at station “S10-1” at a depth of “5” meters.

### DNA extraction and sequencing

The phenol-chloroform-isoamyl alcohol method was applied to extract microbial DNA, as described previously^34^. The quality and concentrations of DNA were quantified using 1% agarose gel electrophoresis and Invitrogen Qubit 2.0 Fluorimeter (ThermoFisher Scientific), respectively. The V4-V5 hypervariable regions of the 16S rRNA gene sequences were amplified using a universal primer pair, 515Y (5′-GTGYCAGCMGCCGCGGTAA-3′) and 926 R (5′-CCGYCAATTYMTTTRAGTTT-3′)^35^. The amplified fragments were sequenced on the Illumina HiSeq 2500 platform using paired-end 2 × 250 bp chemistries as described previously^36^. To ensure data quality, raw reads of 16S rDNA gene sequencing were subjected to adapter trimming and quality control using the cutadapt v4.0 and the fastqc v0.12.1 plugins wrapped in the QIIME2 toolkit suite (version 2022.2)^37^. Amplicon sequence variants (ASVs) and a feature table were generated using the deblur v1.1.1 plugin in QIIME2^38^. The taxonomy of representative ASV sequences was then assigned using the QIIME2 feature-classifier plugin with the pre-trained 99% clustered SILVA database (release 138) as the employed sklearn classifier (Fig. S1).

Qualified DNA samples were fragmented using the Covaris Ultrasonicator M220 (Covaris, USA) with a fragment size of ~500 bp. The resulting DNA fragments were subsequently used in the library preparation and sequencing on an Illumina HiSeq 2500 platform using paired-end 2 × 150 bp chemistries for metagenomic sequencing. All the sequencing jobs were carried out at MAGIGENE (Magigene Biotech, Guangzhou, China).

### Metagenomic assembly, gene annotation and abundance quantification

Raw reads were trimmed and quality filtered using fastp v0.23.1^39^ wrapped in the metaWRAP v1.3 pipeline^40^. Clean reads were assembled using MEGAHIT v1.2.9^41^ with default parameters set by the metaWRAP pipeline. Gene-coding sequences of the assembled contigs were predicted using Prodigal v2.6.3 in “meta” mode^42^. To generate a gene catalog of non-redundant sequences, all the coding sequences were clustered into representative sequences at 95% identity using CD-HT v4.8.1^43^ with parameters: -c 0.95 -d 400 -T 20 -M 20000 -n 5. For each sample, quality-controlled reads were mapped to the non-redundant gene database using bwa v2.2.1^44^, and RPKM (reads per kilobase per million) values were calculated to determine the relative abundance of contigs using coverM v0.3.1 (https://github.com/wwood/CoverM) with parameters: contig mode, --trim-min 0.10 --trim-max 0.90 --min-read-percent-identity 0.95--min-read-aligned-percent 0.75 -m rpkm. Functions of the non-redundant genes were predicted by KofamScan^45^ using the prokaryotic, eukaryotic and viral KEGG gene database (Release 108.1) with default settings (Fig. S1).

### Metagenomic binning

Contigs longer than 1000 bp were grouped into bins using the metaWRAP binning module with three binners: MaxBin2 v2.2.7, MetaBAT2 v2.12.1, and CONCOCT v1.1.0^46–48^. The resulting bins from individual binners were further refined using the bin_refinement module of metaWRAP with >50% completeness and <10% contamination thresholds^37^. In addition, samples were compared using sourmash v4.8.4^49^, and those ones with close community composition were co-assembled and further binned using BASALT v1.0.0^50^ (via MaxBin2 v2.2.7, MetaBAT2 v2.12.1, and CONCOCT v1.1.0 with more-sensitivity parameter)^46–48^ (Fig. S1).

### MAGs refinement and quality assessment

Bins meeting the criteria of ≥50% completeness and ≤10% contamination were subsequently clustered using dRep v3.4.2^51^ at the 95% average nucleotide identity (ANI) threshold (-sa 0.95 -comp 50 -con 10), resulting in a total of 732 species-level bins. The refined bins were further quality checked using CheckM2 v1.0.2^52^ to remove low quality bins, and the remaining 675 bins were classified into high-, medium-quality MAGs according to MIMAG criteria^28^. Taxonomy of each MAG was assigned using GTDB-Tk v2.3.2^53^ based on the Genome Taxonomy Database (GTDB) version r207^31^. In addition, MAGs were functionally annotated using Prokka v1.14.5^54^.

### Phylogenomic tree construction

The 160 and 49 conserved bacterial and archaeal single-copy genes were extracted from these MAGs using GTDB-Tk v2.3.2^53^, respectively. Only marker genes found in ≥30 MAGs were eventually selected to construct the bacterial and archaeal phylogenomic trees. MUSCLE v5^55^ was used to align marker gene sequences extracted from MAGs, and then BMGE^56^ was used to prune the alignments. Phylogenomic trees were constructed using IQTree v2.0.3^57^ with the optimal models (Bacteria: -m Q.pfam + F + I -B 1000, Archaea: -m LG + F + R5 -B 1000) estimated by ModelFinder^58^. The confidence of the maximum-likelihood tree was estimated using 1000 bootstraps.

## Data Records

All sequencing products associated with this project can be found under National Center for Biotechnology Information (NCBI) BioProject ID PRJNA1031568^59^. Clean reads of 16S rDNA amplicon and metagenomic sequencing have been deposited at NCBI with the Sequence Read Archive (SRA) project number SRP468222^60^. NCBI SRA accession numbers for each sample and sequencing type were also provided in Table S1 (Metagenomic Information sheet). Metagenomic assemblies have been deposited at NCBI GenBank database under the same BioProject, and accession numbers can be found in Table S1 (Metagenomic Information sheet). All reads uploaded to the NCBI SRA database were quality-controlled using the software as documented in the “Materials and methods” section. MAGs, Prokka annotations, and function-annotated non-redundant genes with abundance information have been deposited at Figshare^61^. The MAG names were identical to those of the genome bins in Table S2 (MAG information sheet).

## Technical Validation

All raw data processing steps, software, and parameters used in this study were described in the “Materials and methods” section. The assessment of quality scores for the raw reads of the 25 16S rDNA amplicon was performed using FastQC v0.12.1. The results showed that >92.55% of the deduplicated percentage and GC content ≤54%. The assessment of quality scores for the raw reads of the 25 metagenomes was performed using fastp v0.23.1^39^. The results showed that ~95.71% and ~90.14% of the bases have quality scores of ≥20 and ≥30, and GC content <56%, respectively, indicating that sequencing was performed adequately (Table S1). MAGs recovered here were compared with OceanDNA and Tara Oceans using dRep v3.4.2^51^ at the 95% average nucleotide identity (ANI) threshold (-sa 0.95 -comp 50 -con 10) to show the novelty of our MAGs.

### Supplementary information

Supplementary Information

Table S1

Table S2

## Data Availability

All versions of third-party software and scripts used in this study are described and referenced accordingly in the “Materials and methods” section for ease of access and reproducibility.
