# Supplementary material for: Depth-dependent microbial metagenomes sampled in the northeastern Indian Ocean
Source: Sci Data. 2024 Jan 18;11:88. doi: 10.1038/s41597-024-02939-4 (PMC10796761; doi:10.1038/s41597-024-02939-4)
Supplement: Supplementary file 1 — Supplementary Information [file 41597_2024_2939_MOESM1_ESM.docx]

**Supplementary Information**


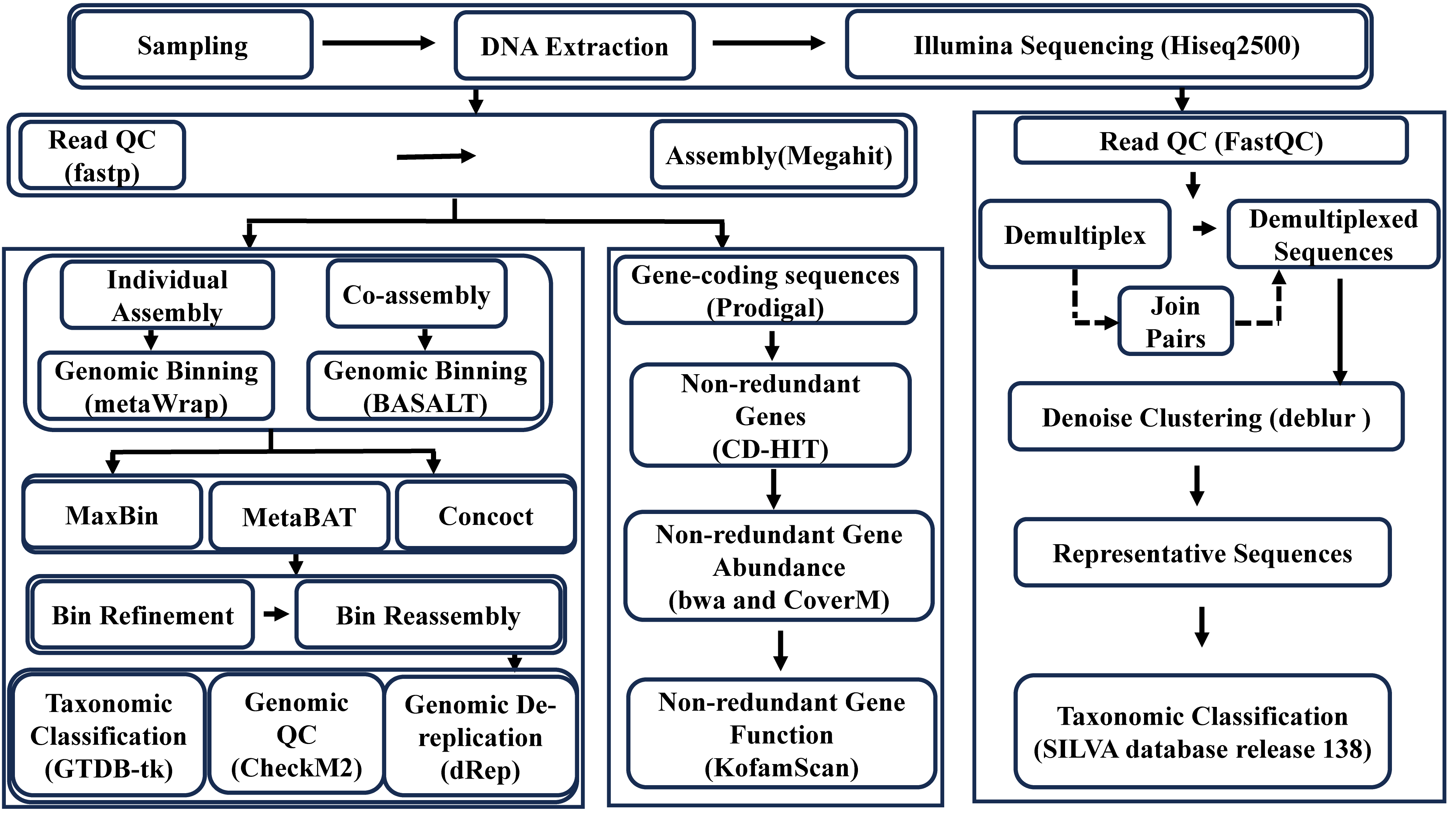


**Figure S1. Bioinformatics workflow for 16S rDNA amplicon and metagenomic data analysis.** After sampling, microbial DNA was extracted and libraries were prepared for 16S rDNA amplicon and metagenomic sequencing. For amplicon data analysis, reads were quality controlled, denoised, and clustered using built-in plugins of the QIIME2. For metagenomic data analysis, a non-redundant gene set was generated via coding gene prediction and dereplication. The preliminary MAGs were obtained after read quality control, metagenomic assembly and binning, then subjected to genome refinement, reassembly, and dereplication. The final MAGs were taxonomically classified and were used for further analysis. A gene catalog was built to include a non-redundant gene set across all samples for all metagenomic contigs. Functional annotation and abundance profiling of these unique genes were also provided. Detailed data processing steps, software, and parameters can be found in the “Materials and methods” section.

**Table S1.** Metadata of samples collected along the E87 transect in the northeastern Indian Ocean.

**Table S2.** MAGs and 16S rDNA ASV table generated in this study.
